# Identification of Pathogenic Genes of Nonsyndromic Hearing Loss in Uyghur Families Using Massively Parallel DNA Sequencing Technique

**DOI:** 10.1155/2018/5298057

**Published:** 2018-03-05

**Authors:** Yu Chen, Yu Lu, Pilidong Kuyaxi, Jing Cheng, Juan Zhao, Qi Zhao, Patiguli Musha, Hua Zhang, Huijun Yuan

**Affiliations:** ^1^Department of Otorhinolaryngology, The First Affiliated Hospital of Xinjiang Medical University, Urumqi 830054, China; ^2^Medical Genetics Center, The First Affiliated Hospital, Army Medical University, Chongqing 400038, China; ^3^Department of Otorhinolaryngology, The First People's Hospital, Kashi Municipality, Xinjiang 844000, China

## Abstract

We aim to identify the mutations of deafness genes using massively parallel DNA sequencing in the 12 Uyghur families. SNPscan method was used to screen against the 124 sites in the common deafness genes in probands. Subjects with SNPscan negativity were subject to massively parallel DNA sequencing for the sequencing of 97 genes known to be responsible for hearing loss. Eight families (66.7%) showed biallelic mutations in probands, including *MYO15A* mutation (*6892C>T* in J02 family, *9514C>T/7894G>T* in J07 family, and *9514C>T* in J16 family), *MYO7A* mutation (*1258A>T* in J03 family), *TMC1* mutation (*773G>A* in J09 family and *1247T>G/1312G>A* in J11 family), and *PCDH15* mutation (*4658delT* in J08 and J13 families). Six novel types of mutation were identified including *6892C>T*, *9514C>T/7894G>T*, and *9514C>T* in *MYO15A* gene, *1258A>T* in *MYO7A*, *773G>A* in *TMC1*, and *4658delT* in *PCDH15*. The ratio of nonsense mutation and frameshift mutation was comparatively high. All these indicated that the mutation types reported in this study were rare. In conclusion, rare deafness genes were identified in the Uyghur families using massively parallel DNA sequencing, part of which were suggested to be related to the pathogenesis of the disease.

## 1. Introduction

Hereditary hearing loss is a highly heterogeneous disease with autosomal recessive nonsyndromic hearing loss (ARNSHL) being the most frequent condition occurring in 70% of the cases and syndromic in the other 30% [1]. Up to now, mutation in *GJB2*, *SLC26A4*, and *12S rRNA* has been considered as the major cause for the Han Chinese with ARNSHL [2, 3].

The genetic etiology of hearing loss may vary in different countries or races. As previously described, the positive rates of common hearing loss genes in the Uyghur minority were significantly lower than those of the Han Chinese (13.06–14.44% versus 32.45–34.05%), indicating that those two ethnicities differed substantially in the mutation spectrum of the common deafness genes [4, 5]. Nowadays, rare studies have been carried out to investigate the genetic etiology of the genes associated with hearing loss in Uyghur minority. In 2015, Chen et al. investigated the nonsyndromic sensorineural hearing loss using targeted next-generation sequencing technique in the Uyghur families, and some novel pathogenic mutations were identified in four probands including the *p.L416R/p.A438T* compound heterozygous mutations in *TMC1*, homozygous *p.V1880E* mutation in *MYO7A*, *c.1238delT* frame-shifting deletion in *PCDH15*, and *c.9690+1G>A* splice site mutation in *MYO15A*. Besides, rare mutations have been identified in the hearing loss that is rarely diagnosed in the Uyghur minority [6]. Xinjiang, officially the Xinjiang Uyghur Autonomous Region, is the largest administrative division in mainland China with about 10 million Uyghur habitants. Therefore, it is necessary to investigate mutations in the relatively rare deafness genes among the Uyghur families. In this study, we recruited twelve recessive Uyghur families that were excluded from mutations in common deafness genes using massively parallel DNA sequencing, to investigate the pathogenic mutations of rare deafness genes.

## 2. Materials and Methods

### 2.1. Subjects

Subjects from 18 Uyghur families received screening from Deaf-Mutes and Disabled Persons' Federation of Kashgar (Xinjiang, China) from March 20, 2014, to November 24, 2015, were recruited in this study. This study was approved by the Ethical Committee of the First Affiliated Hospital of Xinjiang Medical University. Written informed consent was obtained from each subject or their guardians. Eighteen unrelated Uyghur families, with two or more nonsyndromic hearing loss subjects in each family, were included in this study. Those with the possibility of environmental causes or syndromic hearing impairment were excluded from the study. All affected family members were required to receive a complete medical history inquiry and detailed physical examination (i.e., intellectual assessment, ENT routine examination, endoscopy of ear, and auditory threshold test) to exclude the possibility of environmental causes or syndromic hearing impairment. The subjects with hearing impairment were further classified based on their four-frequency (i.e., 0.5, 1.0, 2.0, and 4.0 kHz) pure-tone averages (PTA) into mild (20–40 dB HL), moderate (41–55 dB HL), moderately severe (56–70 dB HL), severe (71–95 dB HL), or extremely severe (>95 dB HL) groups, according to the WHO Hearing Classification International Standard in 2005.

### 2.2. Exception of Common Mutation Responsible for the Congenital Hearing Loss

Venous blood (3–5 mL) was collected from each family member, followed by DNA extraction using the commercial kit (Qiagen, Germany) according to the manufacturer's instructions. SNPscan method was used to prescreen against the 124 sites in the common deafness genes (i.e., *GJB2*, *12S rRNA*, and *SLC26A4*) as previously described [7]. Subsequently, subjects with SNPscan negativity were subject to massively parallel DNA sequencing for the sequencing of 97 genes known to be responsible for hearing loss. All experiments were completed in Genesky Bio-Tech Co. Ltd. (Shanghai, China) at least in triplicate.

### 2.3. Massively Parallel DNA Sequencing

Genome DNA was collected from two subjects with hearing loss in each family using a commercial kit (Agilent Technologies, Santa Clara, CA, USA). The genome DNA library was established according to the manufacturer's instructions, together with the target capture (Agilent Technologies, Santa Clara, CA, USA). All the exons, flanking introns, and splicing regions of the 97 genes were captured. Upon precise quantification, the captured DNA fragments were sequenced on Illumina HiSeq2000 analyzer. Data analysis and bioinformatics processing were performed following standard Illumina procedures.

Reads were aligned to GRCh37/hg19 assembly (https://www.Xncbi.nlm.nih.gov/projects/genome/guide/human/index.shtml) using the BWA software package (http://bio-bwa.sourceforge.net/) to identify the candidate mutations that may affect the function of the protein. Potentially pathogenic variants were defined as nonsense, missense, splice-site, and indel variants with allele frequencies of less than 0.01 in the public databases and the in-house exome database. The candidate pathogenic mutations were genotyped by Sanger sequencing in all family members. Cosegregation was performed to the mutation. The conservation of the target amino acids among the species was analyzed using the ClustalW software (http://www.genome.jp/tools-bin/clustalw).

## 3. Results

### 3.1. Exception of Common Mutation Responsible for the Congenital Hearing Loss

**Figure 1:**
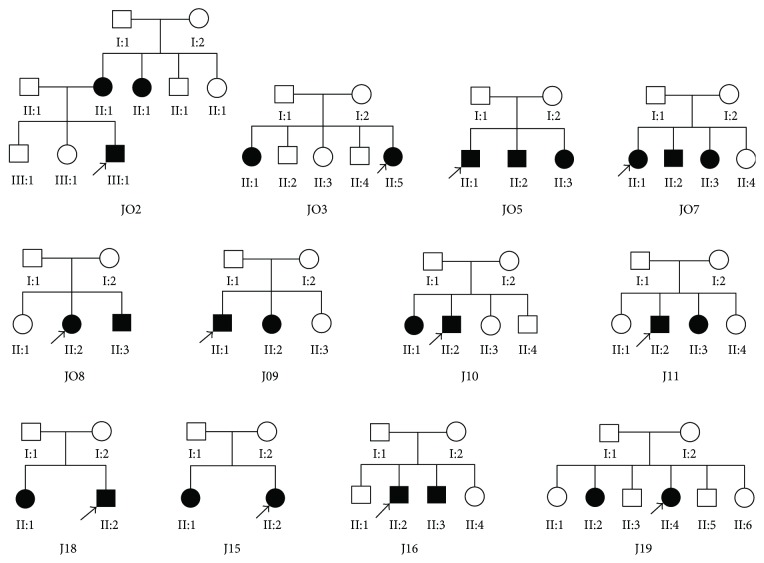
Pedigree of the 12 Uyghur families with hearing loss. Darkened symbols presented patients with deafness. Arrow indicated the probands.

Prescreening of the 124 sites in the *GJB2*, *12S rRNA*, and *SLC26A4* genes was performed in the probands of eighteen Uyghur families. Six families were excluded due to presence of common mutations. There were no common mutations among these probands of the 12 families designated as J02, J03, J05, J07, J08, J09, J10, J11, J13, J15, J16, and J19 families (Figure 1).

### 3.2. Patient Characteristics

Twenty-seven subjects (male: 12; female: 15) with hearing loss from 12 families were finally identified in this study, aging from 3 yrs to 30 yrs. All the subjects were confirmed with severe or extremely severe hearing loss (Table 1). All the family members showed no anomaly except one affected sibling (II:1) in the J03 family showed hearing loss combined with vision impairment and visual field defect. According to the pedigree, the features of hearing loss in all the families were in line with autosomal recessive inheritance.

### 3.3. Identification and Verification of Pathogenic Mutations

**Figure 2:**
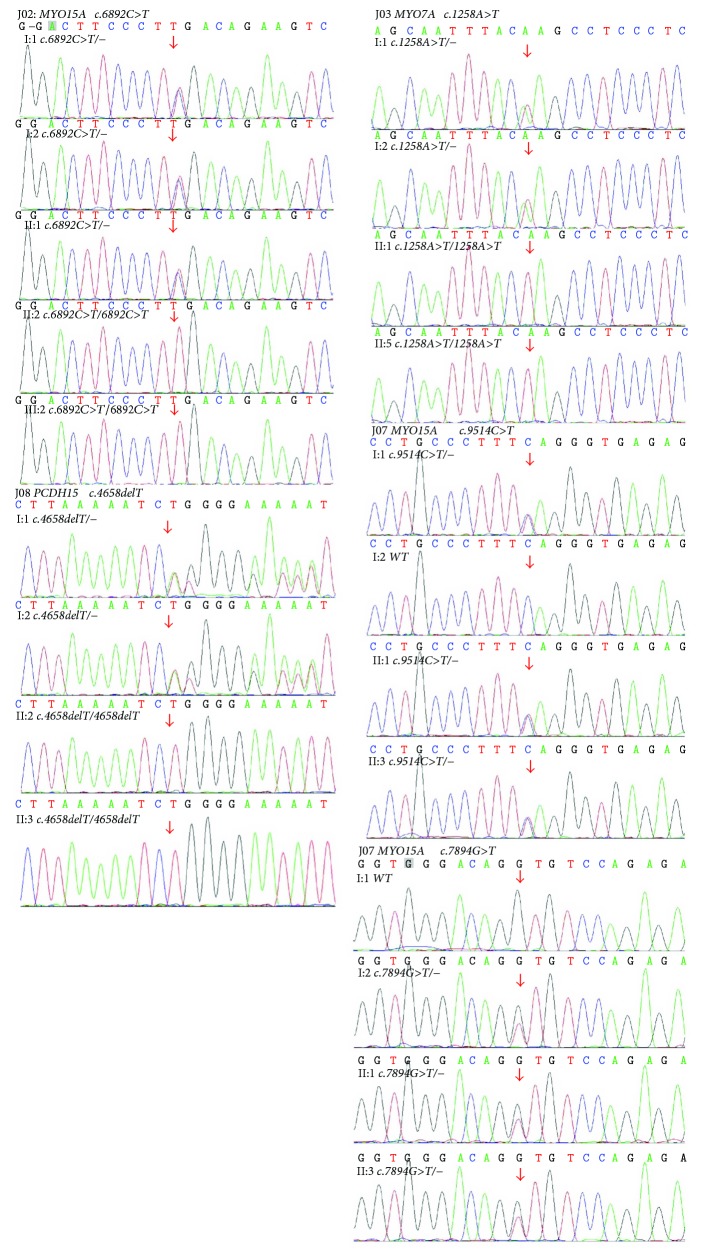
Sequencing results of the J02, J03, J07, and J08 families.

**Figure 3:**
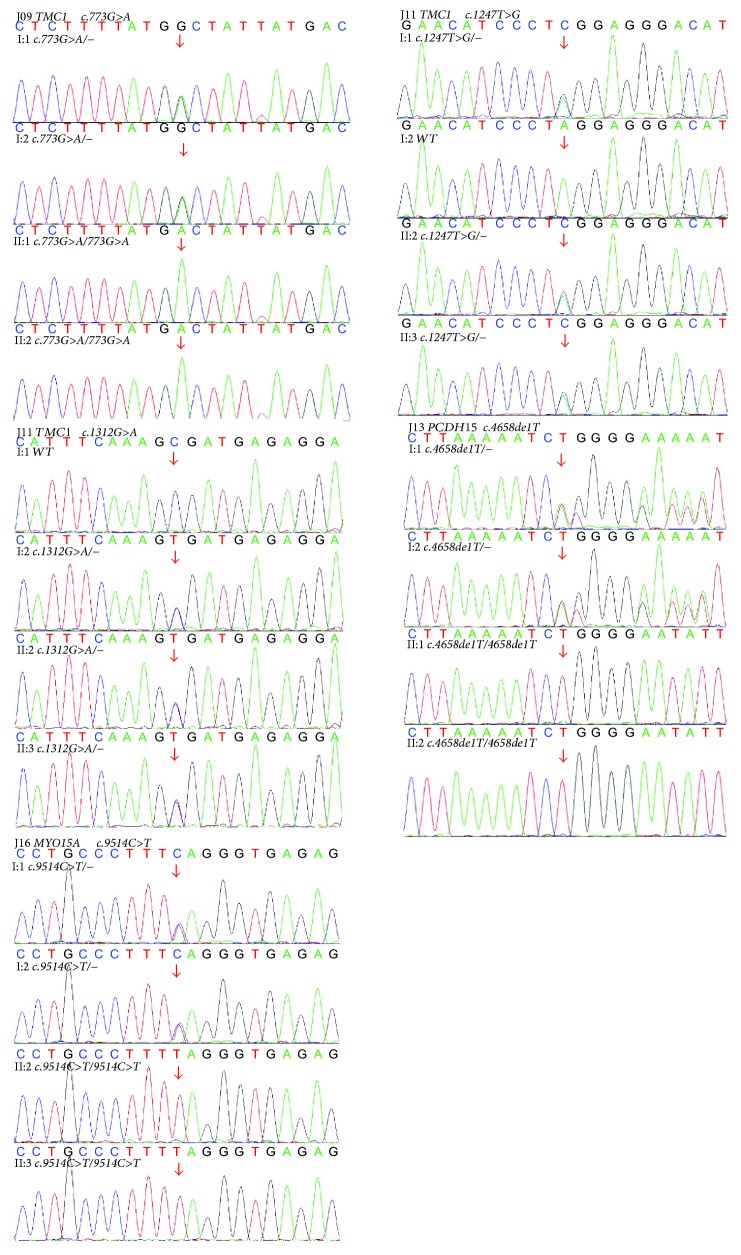
Sequencing results of the J09, J11, J13, and J16 families.

Eight families (66.7%) showed biallelic mutation in the probands (Table 2), including *MYO15A* mutation in three families (i.e., J02, J07, and J16), *MYO7A* mutation in one family (J03), *TMC1* mutation in two families (i.e., J09 and J11), and *PCDH15* in two families (i.e., J08 and J13). Among these families, cosegregation was noticed in the mutation genes among the family members (Figures 2 and 3).

**Figure 4:**
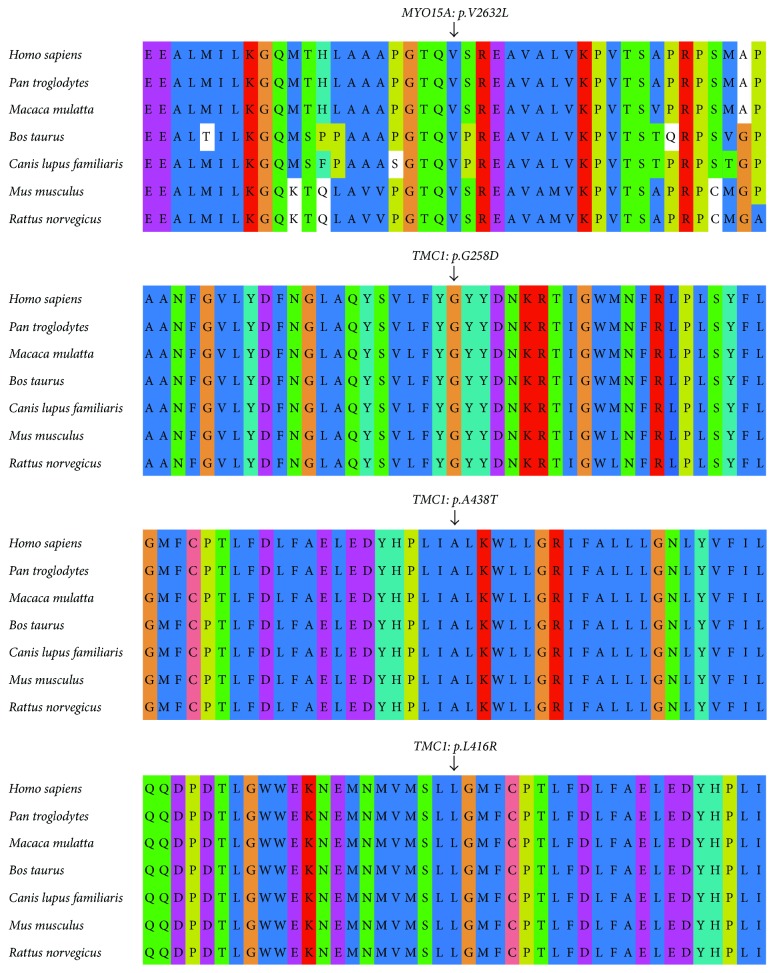
The missense mutations of *p.V2632L* in *MYO15A* and *p.G258D*, *p.L416R*, and *p.A438T* in *TMC1* in multiple species after sequencing.

In the J02 family, homozygous *c.6892C>T* (*p.R2298X*) in *MYO15A* was identified in two subjects (II:2 and III:3). Heterozygous *c.6892C>T* in *MYO15A* was identified in I:1, I:2, and II:1 (Figure 2). In the J03 family, homozygous *c.1258A>T* (*p.K420X*) in *MYO7A* was identified in two siblings (II:1 and II:5). Besides, heterozygous *c.1258A>T* in *MYO7A* was identified in I:1 and I:2 (Figure 2). In the J07 family, compound heterozygous *c.9514C>T* (*p.Q3172X*)/*7894G>T* (*p.V2632L*) in *MYO15A* was identified in two siblings (II:1 and II:3). Heterozygous *c.9514C>T* in *MYO15A* was identified in I:1, and heterozygous *c.7894G>T* in *MYO15A* was identified in I:2 (Figure 2). Meanwhile, homozygous *c.4658delT* (*p.M1553fs*) in *PCDH15* was identified in two siblings (II:2 and II:3) in J08 family and in two siblings (II:1 and II:2) in J13 family, respectively. In their parents (I:1 and I:2) in J08 and J13 families, heterozygous *c.4658delT* was identified in the *PCDH15* (Figures 2 and 3). In the J09 family, the homozygous *c.773G>A* (*p.G258D*) was noticed in the *TMC1* gene in two siblings (II:1 and II:2). Heterozygous *c.773G>A* in *TMC1* was identified in I:1 and I:2 (Figure 3). In the J11 family, the compound heterozygous *c.1247T>G/1312G>A* was identified in the *TMC1* gene in two siblings (II:2 and II:3). Sanger sequencing was performed to their parents (I:1 and I:2), which revealed *c.1247T>G* in I:1 and *c.1312G>A* in I:2 responsible for the *p.L416R* and *p.A438T* mutations, respectively (Figure 3). In J16 family, the homozygous *c.9514C>T* (*p.Q3172X*) was identified in the *MYO15A* in two siblings (II:2 and II:3). Heterozygous *c.9514C>T* in *MYO15A* was identified in I:1 and I:2 (Figure 3). The missense mutations of *p.V2632L* in *MYO15A* and *p.G258D*, *p.L416R*, and *p.A438T* in *TMC1* were highly conserved among various species (Figure 4). No pathogenic mutations were noticed in the other families including J05, J10, J15, and J19, respectively.

## 4. Discussion

Rare studies have been carried out to investigate the molecular etiology of hearing loss in the Uyghur minority as it shows lower incidence compared with that of the Han Chinese [8]. In this study, massively parallel DNA sequencing was used to screen rare deafness genes in the Uyghur families, and several new mutations that were suggested to be related to the pathogenesis of the disease were identified. This study is helpful to increase our understanding on the molecular etiology of hearing loss in Uyghur minority.

Mutations of *MYO15A* at the *DFNB3* locus appear to be the third or fourth most common cause of autosomal recessive nonsyndromic deafness [9]. Myosin 15A is mainly expressed in the cochlea and plays important roles in the differentiation and extension of the stereocilium in the hair cells [10]. Up to now, 48 types of mutation have been reported particularly in the subjects in Pakistan, Turkey, and Iran [8, 11, 12]. In this study, homozygous and heterozygous *MYO15A* mutations were identified in the J02, J07, and J16 families, and their parents were carriers of mutation. In the J02 and J16 families, nonsense mutation was identified in each family, which finally resulted in impairment of protein function and the consequent phenotypes of hearing loss. Nonsense mutation was responsible for extremely severe hearing loss. Our results indicated that subjects with newly identified nonsense mutations (*p.R2298X/p.Q3172*) of *MYO15A* in the J02 and J16 families showed severe or extremely severe hearing loss according to the three-frequency PTA. Additionally, two newly identified mutations (e.g., *p.Q3172X/p.V2632L*) of *MYO15A* were noticed in the J07 family, and the subjects presented severe hearing loss. These results were in line with the fact that *MYO15A* mutation may be associated with severe or extremely severe hearing loss among subjects [13, 14].

*TMC1* encodes a transmembrane protein (TMC1 protein). *TMC1* mutation was reported to induce both autosomal dominant and recessive hearing loss (*DFNA36* and *DFNB7/B11*) in a large number of populations. To date, a total of 52 mutations have been reported in *TMC1* gene [15]. In this study, homozygous mutation and compound heterozygous mutation were identified in the *TMC1* gene in the J09 and J11 families, with the parents as the mutation carriers. To be exact, missense mutations (*p.G258D/p.G258D* and *p.L416R/p.A438T*) were identified in the J09 and J11 families. These sequence mutations in amino acid were highly conserved among the species. On this basis, we speculated that the mutations may induce function loss of the encoded proteins, which affects the ion channel formation on the surface of the hair cells in the internal ear. The transmission of the potassium ion was hampered, which consequently led to dysfunction of cochlear hair cells [16]. Among these mutations, *p.L416R/p.A438T* compound heterozygous mutations in *TMC1* were considered as pathogenic mutations in Uyghur families [6]. A novel *p.G258D* was identified in one family in this study. Autosomal recessive hereditary deafness caused by *TMC1* gene was mainly featured by severe or extremely severe congenital sensorineural deafness. In line with the previous study, the patient showed clinical manifestations of severe congenital deafness in the J09 family, while the patient showed manifestations of extremely severe congenital deafness in the J11 family. Whereas, some patients may present phenotype of severe sensorineural hearing loss [17, 18].

Mutations of *PCDH15*, encoding protocadherin 15, are responsible for inducing combined hearing and vision impairment (type 1 Usher syndrome; USH1F) or nonsyndromic deafness (DFNB23) [19]. Human PCDH15 is expressed in the cochlea, the external synapse of the optic nerves, and the retinal cells. To the best of our knowledge, *PCDH15* mutation could induce nonsyndromic hearing loss and Usher syndrome type 1F. The major difference between these conditions is the presence of vision loss [20]. The relationship between the genotype and phenotype of *PCHD15* was closely related to the mutation type [21]. For example, frameshift mutation or nonsense mutation may induce syndromic hearing loss. For the missense mutation, the hearing rather than visual acuity may be hampered due to mutation, which finally lead to syndromic hearing loss [22, 23]. In this study, a newly identified frameshift mutation (*p.M1553fs*) was noticed in the J08 and J13 families. We speculated that it might induce structural changes of encoding protein together with tip link of the hair cells in the inner ear and finally lead to hearing loss. The patients (aged 12–22 yrs) showed severe or extremely severe hearing loss. Despite frameshift mutations were identified in these patients, no visual disorder featured by night blindness was observed with the aging of the patients. Besides, in a consanguineous Pakistani family, missense mutation of *PCDH15* was reported to be responsible for inducing Usher syndrome IF type. On this basis, the relationship between phenotype and genotype of *PCDH15* is still not well defined. Further observations should be paid to the occurrence of delayed visual disorder in the patients of J08 and J13 families.

The *MYO7A* gene encodes the actin-binding motor protein myosin VIIa. The myosin VIIa protein is expressed in the cochlea, cytoplasm of outer hair cells, stereocilium, retinal pigment epithelial cells, photoreceptor cells, and epithelial cells on the vestibular nerves [24]. To date, more than 160 different mutations of *MYO7A* gene have been identified, most of which have been reported to induce Usher syndrome type IB and nonsyndromic Usher syndrome [25, 26]. Part of the mutations has been reported to be associated with nonsyndromic hearing loss (DFNAII and DFNB2) [25]. On this basis, it is reasonable to speculate that there might be sharp differences in the variance of phenotype after mutation. Similar with *PCDH15*, the difference of syndromic and nonsyndromic hearing loss induced by mutation of *MYO7A* was visual disorder. In cases of *MYO7A* mutation, the function of MYO7A protein in the retina may be compensated by the protein with similar function or by the residue wild-type dimer. Otherwise, vision loss may be induced as no adequate compensation of the protein function in the retina by the wild-type dimer. Nevertheless, the function of MYO7A protein could not be compensated in the internal ear, which led to functional loss [26].

USH1B and USH1F were featured by constriction of visual field, visual disturbance with or without vestibular dysfunction caused by progressive retinitis pigmentosa [27, 28]. In this study, a novel nonsense mutation (*p.K420X*) was noticed in *MYO7A* in the J03 family, which may induce hearing loss by changing the protein structures. Two siblings (II:5, 6 yrs, with no visual disorder; II:1, 17 yrs) in the family showed severe congenital hearing loss. For II:1, the patient presented visual disorder at the age of 7, which showed gradual deterioration together with obvious light blindness and spot-like defect of visual field. No vestibular disorder was noticed. For the II:5, no visual disorders were noticed as the patient was at the young age. The phenotypes of the II:1 and II:5 were different despite the same mutation in *MYO7A*, which were manifested as DFNB2 and USH1B, respectively. In the future, close observations should be paid to the visual disorder of the II:5 to confirm whether she suffers from USH1B or not.

Indeed, there are some limitations in this study. Actually, the sample size is not large enough. In addition, we are not sure whether the identified mutations can represent the whole Uyghur population with hearing loss in mainland China. In the future, we will focus on large sample studies to identify the roles of these mutations in the pathogenesis of hearing loss.

In conclusion, mutations in rare hearing loss genes were noticed in 8 Uyghur families using massively parallel DNA sequencing. In total, 6 novel types of mutation were identified including *6892C>T*, *9514C>T/7894G>T*, and *9514C>T* in *MYO15A* gene (J02, J07, and J16 families), *1258A>T* in *MYO7A* (J03 family), *773G>A* in *TMC1* (J09 family), and *4658delT* in *PCDH15* (J08 and J13 families). Besides, the ratio of nonsense mutation and frameshift mutation was comparatively high, which indicated that the mutation types were rare. The homozygous mutations in the Uyghur families were higher in incidence. Our study contributed to the investigation of molecular etiology of hearing loss in Uyghur minority.

## Figures and Tables

**Table 1:** General information and severity of deafness in the families.

Family	Patients with deafness	Gender	Age (yrs)	Hearing loss
J02	II:2	Female	30	Extremely severe
	II:3	Female	26	Severe
	III:3	Male	6	Extremely severe

J03	II:1	Female	17	Extremely severe
	II:5	Female	3	Extremely severe

J05	II:1	Male	18	Extremely severe
	II:2	Male	12	Extremely severe
	II:3	Female	9	Extremely severe

J07	II:1	Female	20	Extremely severe
	II:2	Male	19	Extremely severe
	II:3	Female	13	Severe

J08	II:2	Male	16	Severe
	II:3	Female	12	Extremely severe

J09	II:1	Male	20	Severe
	II:2	Female	12	Severe

J10	II:1	Female	14	Severe
	II:2	Male	12	Severe

J11	II:2	Male	17	Extremely severe
	II:3	Female	11	Extremely severe

J13	II:1	Female	22	Severe
	II:2	Male	18	Severe

J15	II:1	Female	16	Extremely severe
	II:2	Male	13	Extremely severe

J16	II:2	Male	19	Severe
	II:3	Male	15	Severe

J19	II:2	Female	17	Severe
	II:4	Female	11	Severe

**Table 2:** Type of mutations in the families.

Type	Family	Mutation site	Amino acid changes	Type of mutation	Novel mutation
*MYO15A*	J02	*6892C>T*	*p.R2298X*	Nonsense mutation	Novel
	J07	*9514C>T/7894G>T*	*p.Q3172X/p.V2632L*	Nonsense mutation/missense mutation	Novel
	J16	*9514C>T*	*p.Q3172X*	Nonsense mutation	Novel

*MYO7A*	J03	*1258A>T*	*p.K420X*	Nonsense mutation	Novel

*TMC1*	J09	*773G>A*	*p.G258D*	Missense mutation	Novel
	J11	*1247T*>*G/1312G>A*	*p.L416R/p.A438T*	Missense mutation	Reported previously

*PCDH15*	J08	*4658delT*	*p.M1553fs*	Frameshift mutation	Novel
	J13	*4658delT*	*p.M1553fs*	Frameshift mutation	Novel

## Abbreviations

ARNSHL: Autosomal recessive nonsyndromic hearing loss
PTA: Pure-tone averages.
